# Hybrid FSK–FDM Scheme for Data Rate Enhancement in Dual-Function Radar and Communication

**DOI:** 10.3390/s23125440

**Published:** 2023-06-08

**Authors:** Muhammad Fahad Munir, Abdul Basit, Wasim Khan, Athar Wasim, Muhammad Mohsin Khan, Ahmed Saleem, Salman A. AlQahtani, Amil Daraz, Pranavkumar Pathak

**Affiliations:** 1 Department of Electrical & Computer Engineering, International Islamic University, Islamabad 44000, Pakistan; fahad.munir@iiu.edu.pk (M.F.M.); athar.waseem@iiu.edu.pk (A.W.); ahmed.phdee11@iiu.edu.pk (A.S.); 2 Department of IT & CS, Faculty of FECID, PAF-IAST, Haripur 22620, Pakistan; mohsin.khan@spcai.paf-iast.edu.pk; 3 Department of Computer Engineering, College of Computer and Information Sciences, King Saud University, P.O. Box 51178, Riyadh 11543, Saudi Arabia; salmanq@ksu.edu.sa; 4 School of Information Science and Engineering, NingboTech University, Ningbo 315100, China; 5 School of Continuing Studies, McGill University, QC H3A 0G4, Canada; pranavkumar.pathak@mail.mcgill.ca

**Keywords:** spectrum sharing DFRC, FSK–FDM DFRC, DFRC information embedding, DFRC waveform design, waveform diversity, frequency diversity, joint waveform and frequency, frequency division multiplexing

## Abstract

In this paper, we present a hybrid frequency shift keying and frequency division multiplexing (i.e., FSK–FDM) approach for information embedding in dual-function radar and communication (DFRC) design to achieve an improved communication data rate. Since most of the existing works focus on merely two-bit transmission in each pulse repetition interval (PRI) using different amplitude modulation (AM)- and phased modulation (PM)-based techniques, this paper proposes a new technique that doubles the data rate by using a hybrid FSK–FDM technique. Note that the AM-based techniques are used when the communication receiver resides in the side lobe region of the radar. In contrast, the PM-based techniques perform better if the communication receiver is in the main lobe region. However, the proposed design facilitates the delivery of information bits to the communication receivers with an improved bit rate (BR) and bit error rate (BER) regardless of their locations in the radar’s main lobe or side lobe regions. That is, the proposed scheme enables information encoding according to the transmitted waveforms and frequencies using FSK modulation. Next, the modulated symbols are added together to achieve a double data rate using the FDM technique. Finally, each transmitted composite symbol contains multiple FSK-modulated symbols, resulting in an increased data rate for the communication receiver. Numerous simulation results are presented to validate the effectiveness of the proposed technique.

## 1. Introduction

Spectrum-sharing techniques have received substantial attention from the research community to meet the high data rate demands of the users for 5G and beyond applications [1,2,3]. Modern technologies are prevalent today, such as smartphones, autonomous vehicles, wearable devices, etc. These technologies have made it easier for people to communicate, access information, and move around. They are changing the way we live, work, and interact with the world [4]. In response to the increased demand for the same spectrum, congestion has arisen. Due to this crisis, radar and communications systems, traditionally designed and developed independently, have emerged as a unified or compatible system [5]. A solution to the problem of two or more systems sharing the RF spectrum simultaneously has been proposed in the literature known as spectrum sharing. Therefore, to cope with 4G and 5G services, frequency sharing has become an increasingly critical issue for communication devices [6]. This has encouraged researchers to explore more innovative ways to maximize the available spectrum. Due to its massive allocation of frequencies, the radar spectrum is a viable candidate for frequency sharing.

Note that radar applications, nowadays, are not limited to monitoring purposes only. They are used for geophysical checking, weather monitoring and forecasting, air traffic surveillance, etc. [7]. Although the frequency spectrum (e.g., between 1 to 10 GHz) was originally divided between radars and communication system applications, a significant percentage of these frequencies are distributed among radars. Moreover, high frequencies, e.g., millimetre wave band frequencies, benefit communication system designs to achieve high data rates. Still, these are also used for radar systems as well for improved detection and tracking performances. More importantly, designing a hybrid radar and communication system in necessary to meet this new era’s requirements. On the other hand, mutual interference issues may arise, concerning both military and civilian applications. However, due to rapid growth in the cellular sector, today’s challenge is to maintain a quality service with higher data rates. For improved joint radar and communication (JRC) designs, all the aforementioned challenges need to be addressed, including identifying wireless broadband frequency bands.

According to studies [8,9,10,11], GSM systems (GPRS, EDGE) can interfere with UHF radars operating in the L band between 1 and 2 GHz; whereas, in the S-band, long-term evolution (LTE) and WiMax overlap with airport surveillance or air traffic control (ATC) radar with frequencies between 2 and 4 GHz. Moreover, WiMax and radar overlaps are also mentioned in [12,13,14]. It is also worth noting that millimetre waves, used for orthogonal frequency division multiplexing (OFDM), single carrier, and WLAN, ranging from 11 to 33 ft, used for indoor communication, overlap with high-resolution imaging radar systems. Similarly, the same OFDM-based wireless LAN (WLAN) commonly used for outdoor activities, with ranges between 100 m and 5 km, overlaps with weather radars operating between 2 and 4 GHz in the C band. In order to make wireless communication more efficient, efforts must be made to devise a way of sharing the spectrum to meet extra bandwidth needs. This will benefit us economically, politically, and socially in the near future [13]. Recently, dual-function radar and communication (DFRC) designs have been proposed to share the radar spectrum with communication designs [15]. Generally, the radar operations are performed primarily in a DFRC system, whereas the communication operations are secondary [4,16,17]. Note that the efficient collaboration of the radar and communication systems is required to provide diverse spectrum-sharing methods to meet the data rate requirements for the desired services [18]. Moreover, a few innovative designs and techniques, e.g., cognitive radios and radars [19,20], may open new horizons in terms of usage and efficiency.

Note that radar and communication design sharing can be achieved initially in terms of time and frequency. In time-based sharing, a strobe switch allocates slots to radar and communication receivers [21]. Contrary to the aforementioned method, the spectrum-sharing-based approach provides opportunities simultaneously, leading to an emerging research domain named communication and radar spectrum sharing (CRSS) or integrated sensing and communication (ICAS). Two broad categories can be identified within this research field, i.e., joint radar and communication coexistence (JRCC) and dual-function radar and communication (DFRC). In JRCC, the radar and communication designs use their own transmitting hardware and only share the available frequency spectrum. A radar system of this type is also called an opportunistic system, in which the radar holds the primary position while the communication takes on secondary roles. In addition to coexistence, cooperation, and co-design, JRCC can be divided into three subcategories [22]. Coexistence setups mitigate interference without exchanging information. In contrast, the primary objective of a cooperation setup is to explicitly share information with the beneficiaries, such as radars and communication systems [23].

On the other hand, the DFRC design uses a suitable transmitter that simultaneously facilitates both the radar and communication system in terms of hardware and frequency spectrum, where the system performance is improved by using shared knowledge. More importantly, the spectrum in the DFRC design can be shared with the wireless communication system by three well-known methods, i.e., (a) radar waveform, (b) communication waveform, and (c) multi-beam methods [1]. As radar is a primary operation, the information bits are generally embedded into radar-based waveforms [16]. However, all these techniques require further investigation to improve performance, including waveform diversity with reduced hardware costs for efficient spectrum utilization [24].

The embedding of the information bits into the radar-based waveforms using time-modulated arrays is presented in [25], where the information is encoded in the side lobe levels (SLLs) of the radiated radar beam pattern. Next, the information is decoded at the communication receiver by computing the received power level. Although this SLL-based design is easy to implement, the data rate is significantly low. To make an improved and secure transmission, amplitude shift keying (ASK) has been proposed for information embedding towards communication receivers located in the side lobe regions of the radar beam [26]. The main disadvantage of the ASK-based modulation is its poor performance and bit rate deterioration if both the radar and communication receivers reside at the main lobe of the radar beam pattern. Fortunately, a phase shift keying (PSK)-based technique has been proposed to overcome this issue [25]. The PSK-based technique performs well for the communication receiver located in the main lobe region of the radar beam. The authors of [27] claimed that the PSK-based method is more secure than the ASK method because interference can disintegrate the SLL compared to the phases of the waveform. Unfortunately, the overall communication system performance drastically deteriorates when amplitude modulation-based bit embedding acts in the main lobe region and phase modulation acts in the side lobe regions, respectively. This motivates us to find a method suitable for both the main lobe and side lobe-based communication directions. Furthermore, the QAM-based information embedding was discussed in [28], where SLL and waveform diversity were efficiently controlled, claiming performance efficiency compared to the existing ASK- and PSK-based techniques. For more information on existing information embedding techniques utilizing radar-based waveforms, communication-based waveforms, and sub-beam sharing techniques, readers may refer to [1] for more details.

This paper presents a new approach to embedding information in radar waveforms by a hybrid FSK–FDM technique, which enjoys the benefits of both the modulations and multiplexing techniques. Note that the AM-based techniques are used when the communication receiver resides in the side lobe region of the radar. In contrast, the PM techniques perform better in communication receivers in the main lobe region. However, the proposed design facilitates the delivery of information bits to the communication receivers with an improved bit rate (BR) and bit error rate (BER) regardless of their locations in the radar’s main lobe or side lobe regions. Initially, a lookup table containing the symbols mapped against the possible combination of two information bits is maintained. Since each symbol contains information about the orthogonal waveforms and frequencies, a composite signal is generated by adding multiple symbols using a linear adder. Eventually, the overall data rate significantly increased when adding the symbol through a linear adder in each PRI. Next, this composite signal is modulated at an intermediate frequency upon which the radar operates. Finally, the received signal at the communication receiver is passed through the bandpass filtering procedure to extract the individual symbol. Each combination of waveform and frequency is decoded using matched filtering. The main attributes of this paper are summarized as follows:

- The proposed design facilitates the delivery of information bits to the communication receivers with an improved bit rate (BR) and bit error rate (BER) regardless of their locations in the radar’s main lobe or side lobe regions.
- The proposed method offers reduced inter-symbol interference as the decoding of each composite symbol at any communication receiver is independent of other neighbouring symbols.

The rest of the paper is organized as follows. Section 2 explains the signal data model, while Section 3 presents the proposed information embedding approach at the transmitter side. Furthermore, performance analysis at the receiver is provided in Section 4, while Section 5 presents the simulation results, followed by the conclusion in Section 6.

## 2. Signal Data Model

A signal data model is developed in the following sections for the DFRC design, using FSK and FDM techniques. The DFRC transmitter, the radar receiver, and the communication receiver are all equipped with uniform linear arrays (ULAs) using $M_{T}$, $M_{R}$, and $N_{R}$ antenna elements, respectively. In general, all arrays maintain a half-wavelength spacing between the elements. In this paper, it is considered that the DFRC transmitter and radar receiver are placed so close to each other that they receive the same angle of radiation from each other. The transmitter array mainly generates pulses for detecting and tracking radar targets. The secondary objective of the transmitter array is to embed communication bits in the pulses without affecting the radar operation. The DFRC transmitting array steers the transmitted power within the main beam, where radar operation occurs. The $M_{T}\times 1$ vector form of the baseband signal for the τth radar pulse at the input of the transmit antenna [29] is

(1) $$s\left(t,\tau \right)=\lambda \left(\tau \right)w^{*}\left(t\right)x_{fdm}\left(t\right)$$

where time within each radar pulse is represented by *t*, and the pulse number is represented by τ. For each transmitted waveform, λ(t) determines how much power is assigned to each waveform such that the total transmitted power is fixed. This vector is primarily designed to focus the transmitted power inside the main beam of the radar while minimizing the power radiated outside the main beam. Similarly, w(t) represents the uniform transmit array beam-forming weight vector with dimensions $M_{T}\times 1$ for all waveform combinations, ${\left(.\right)}^{*}$ denotes the complex conjugate and $x_{fdm}\left(t\right)$ is the composite vector developed by adding multiple FSK-modulated symbols. Each FSK symbol represents two bits of information based on multiple orthogonal waveform combinations. More details about the construction of $x_{fdm}\left(t\right)$ are discussed in Section 3. It is assumed that the proposed waveform vectors must be orthogonal in order to be effective, but this is not necessarily true of the baseband signals s(t,τ).

## 3. Proposed Transmit Signalling Strategy for Information Embedding

**Figure 1:**
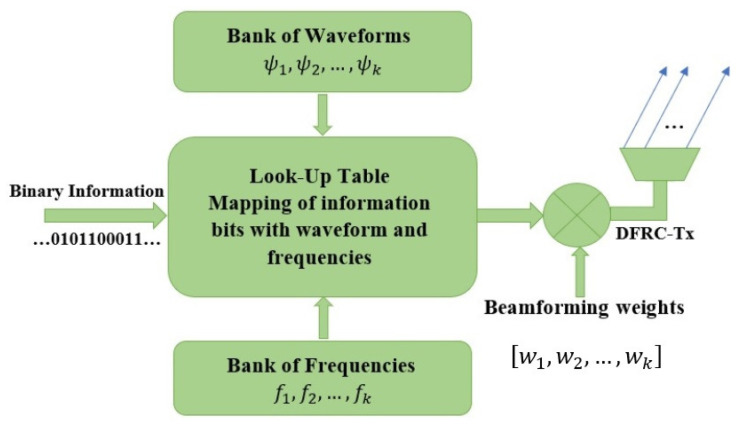
The proposed information embedding methodology at the DFRC transmitter.

Binary information is embedded in radar signals in the form of waveform numbers and frequencies. These signals are sent from a DFRC transmitter and received by both a radar and communication receiver. The data is then extracted from the signal, allowing the radar receiver to determine the objects’ direction and velocity. Two bits of information are mapped during each radar transmit pulse, whereas two frequencies are used in this technique to represent either binary 0 or binary 1, as shown in Figure 1.

The two frequencies are selected from a pool of available frequencies, depending on the information the transmitter wants to communicate. The communication receiver then decodes the frequencies and extracts the binary information from the signal. Similarly, the two waveforms represent binary 0 or binary 1. This information mapping is performed through FSK. This method is based on the fact that different frequencies can be easily distinguished and used to represent different binary values. In FSK, two different frequencies are assigned to represent the binary digit 0 and digit 1. The transmitter then sends these frequencies, one after the other. The receiver decodes the frequencies to extracts the binary data from the signal. Thus, employing FSK modulation, two bits can be represented by distinct frequencies and waveforms, as shown in Table 1.

Each communication symbol encodes two bits of information using a waveform and frequency combination. Consider the random bits pattern shown in Table 2. By mapping each bit pattern to a specific waveform and frequency combination, it is possible to construct two distinct symbols that can be sent over a communication channel. These symbols can then be decoded back to the corresponding bit pattern on the receiver side. The pattern selector selects a suitable combination of waveform and frequency to be transmitted.

**Figure 2:**
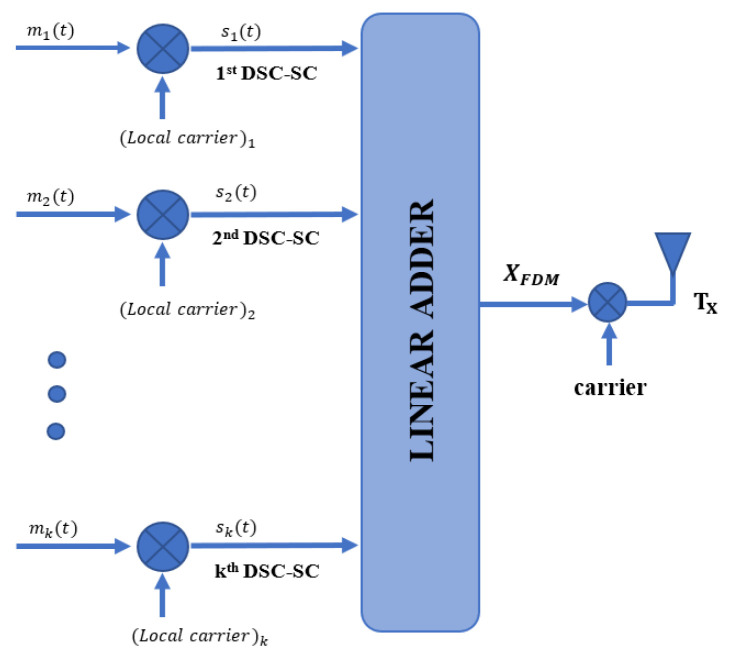
The proposed composite information embedding methodology using frequency division multiplexing at the DFRC transmitter.

For example, for $B_{l}\left(t\right)=00$, the joint waveform and frequency (JWF) combination of $\xi_{00}\left(t\right)$ has been selected from the lookup table. Moreover, multiple JWF combinations are added to make a composite signal using FDM. This signal is then transmitted from the DFRC transmitter as shown in Figure 2. Next, the modulated symbols are added together to achieve a double data rate using the FDM technique. The composite signal is received by the communication receiver, where the JWF symbols are separated via a frequency demodulator. The pattern selector then decodes these JWF combinations into the corresponding bit patterns. These bit patterns can then be converted back to the symbols originally sent by the transmitter. The symbols are then converted to binary data, which can be used for further processing. The mathematical description of a composite signal is given as:

(2) $$x_{fdm}\left(t\right)=\sum_{j=1}^{J}m_{j}\left(t,\tau \right)$$

where, $m_{j}$, j=1,…,J is the JWF symbol added together to double the data rate in the proposed scheme. The overall form of the JWF sample with allocated power can be written as

(3) $$m_{i,j}\left(t\right)=\sqrt{\frac{M_{T}}{L_{B}}}\left(t\right)\xi_{i,f_{j}}\left(t\right)$$

The nomograph of the proposed FDM-based composite signals with linear adder is shown in Figure 2.

Note that all waveforms must be mutually orthogonal for improved communication with less inter-waveform interference.

## 4. Receiver Design

In this section, the radar and communication receivers are discussed with respect to the DFRC transmitter in detail.

### 4.1. Radar Receiver

Assume that the radar main beam contains *M* far-field targets. The vector form of the baseband signal received by the radar receiver is expressed as

(4) $$x_{r}\left(t,\tau \right)=\sqrt{\frac{M_{T}}{L_{B}}}\sum_{m=1}^{M}\beta_{m}\left(a^{T}\left(\theta_{m}\right)s\left(t,\tau \right)\right)b\left(\theta_{m}\right)+e_{r}\left(t,\tau \right)+n_{r}\left(t,\tau \right)$$

where $\sqrt{\frac{M_{T}}{L_{B}}}$ is the received signal power, $\beta_{m}$ is the reflection coefficient of the *m*th target, $a(\theta_{m})$ is the steering vector in the direction $\theta_{m}$ from the dual-function transmitter, s(t,τ) is the base band signal, $b(\theta_{m})$ is the steering vector in the direction $\theta_{m}$ at the receiver, $e_{r}\left(t,\tau \right)$ is the interference vector at the radar receiver, and $n_{r}\left(t,\tau \right)$ is the AWG noise vector zero mean with variance $\sigma^{2}I$ at the radar receiver.

The reflection constant, $\beta_{m}$, remains constant during each pulse but varies on a pulse to pulse basis, obeying the Swerling II model. Similarly, $e_{r}\left(t,\tau \right)$ is the interference vector that impinges on the receiver array from the side lobes. It is important to note that processing is performed directly on the receiver array $M_{T}\times 1$ vector $x_{r}\left(t,\tau \right)$ without going into waveform diversity at this stage.

### 4.2. Communication Receiver

There are *K* communication receivers in the far field, each having an array of $N_{R}$ elements. For ease and convenience and a priory communication, the lookup table containing the dictionary of the orthogonal JWF made using FSK modulation and FDM symbols at the dual-function transmitter is known to each communication receiver. Assume that the *k*th communication receiver, equipped with $N_{R}$ antenna elements arranged uniformly in a linear shape, receives the following FSK–FDM composite signal.

(5) $$y_{k_{fdm}}\left(t,\tau \right)=\sqrt{\frac{M_{T}}{L_{B}}}\alpha_{k}\left(a^{T}\left(\phi_{i}\right)s\left(t,\tau \right)\right)c_{k}\left(\phi_{i}\right)x_{fdm}\left(t,\tau \right)+n_{k_{fdm}}\left(t,\tau \right)$$

where $\sqrt{\frac{M_{T}}{L_{B}}}$ is the received power at the communication receiver, $\alpha_{k}$ is the channel coefficient constant from the transmitter array towards the *k*th communication receiver which summarize the propagation environment, $a(\phi_{i})$ is the steering vector in the direction $\theta_{m}$ from the dual-function transmitter, s(t,τ) is the base band signal, $c_{k}\left(\phi_{k}\right)$ is the steering vector from the receive array in the direction $\phi_{k}$ from the communication receiver, $x_{fdm}$ is the composite FSK–FDM signal, $n_{k}\left(t,\tau \right)$ is the AWG noise vector zero mean with variance $\sigma^{2}I$ at the communication receiver, and $\left(\phi_{k}\right)$ is the direction of the *k*th communication receiver.

As a first step, the beam-forming operation is applied to the received signal. The steering vector is separated from it by multiplying the beam-forming weights at the communication receiver. This allows for the extraction of the desired signal from the received signal, allowing the receiver to focus on the direction of the signal and ignore signals from other directions. This reduces interference from other sources and improves the signal-to-noise ratio (SNR) at the receiver. The beam-forming operation is mathematically expressed by Equation (6).

(6) $$g_{k_{fdm}}\left(t,\tau \right)=c_{k}^{H}\left(\phi_{j}\right)y_{k_{fdm}}\left(t,\tau \right)$$

**Figure 3:**
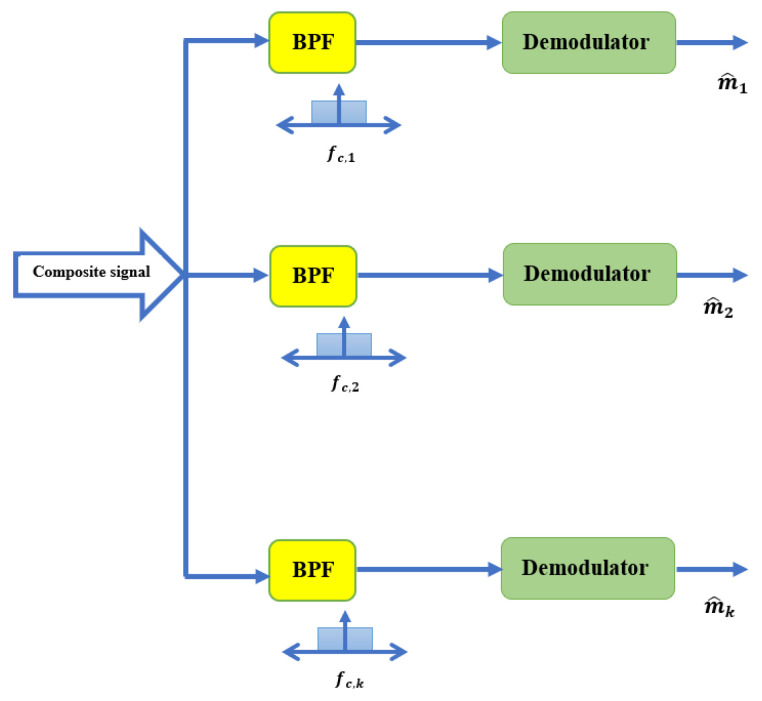
The proposed composite information decoding methodology at the communication receiver.

The next step in this process is to apply bandpass filtering techniques to the received FDM composite signal. The bandpass filter is implemented as a digital finite impulse response (FIR) filter and configured to have a passband with the desired bandwidth. The filtered signal is then demodulated to recover the original transmitted information. A mathematical description is given in Equation (7) and a graphic representation is shown in Figure 3.

(7) $$r_{k}\left(t,\tau \right)=v\left(\Omega \right)g_{k_{fdm}}\left(t,\tau \right)$$

where v(Ω) is the bandpass filtering coefficients at the *k*th communication receiver.

Matched filtering is then applied to the signal, Equation (7), to identify the actual binary information transmitted. This involves multiplying the signal by a reference signal delayed by the same amount of time as the original signal. The multiplication results are then accumulated over a period of time. The accumulated signal can be used to identify the binary information transmitted. Matched filtering is mathematically described by Equation (8).

(8) $$y_{k,l}\left(t,\tau \right)=\left\{\begin{array}{c} \sqrt{\frac{M_{T}}{L_{B}}}r_{1}\left(t,\tau \right)+n_{1}\left(t,\tau \right),\;if\;B_{l}=00\\ \sqrt{\frac{M_{T}}{L_{B}}}r_{2}\left(t,\tau \right)+n_{2}\left(t,\tau \right),\;if\;B_{l}=01\\ \sqrt{\frac{M_{T}}{L_{B}}}r_{3}\left(t,\tau \right)+n_{3}\left(t,\tau \right),\;if\;B_{l}=10\\ \sqrt{\frac{M_{T}}{L_{B}}}r_{4}\left(t,\tau \right)+n_{4}\left(t,\tau \right),\;if\;B_{l}=11 \end{array}\right.$$

**Figure 4:**
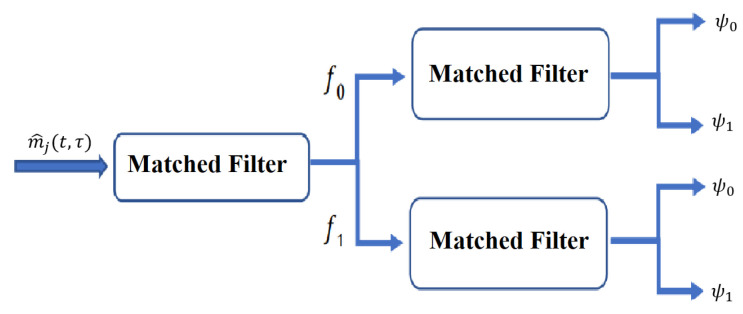
Detailed demodulator design in terms of matched filtering after the bandpass filter at the communication receiver.

The overall process of match filtering and information decoding at the communication receiver is shown in Figure 4.

Furthermore, by performing the simple ratio test on the output of the filter in Equation (6), we obtain

(9) $$\hat{B_{l}}\left(\tau \right)=\left\{\begin{array}{c} 0\;if\;f_{0}\left(t\right),\;\left|y_{k,l}\right|⩾T\\ 1\;if\;f_{1}\left(t\right),\;\left|y_{k,l}\right|⩽T \end{array}\right.$$

where *T* is the threshold constant of frequency separation for orthogonality.

It is important to note that multiple waveforms are selected at a time, and they change on a pulse-to-pulse basis. The data rate is given as a product of the number of bits per pulse and the PRF, i.e.,

(10) DataRate=PRF×bitspercompositesymbol.

The probability that 00 is transmitted and 01 is received can be written as P(01|00). Similarly, when 10 is received, it can be written as P(10|00) and the overall equation with error function can be modelled as

(11) P(00|00)=1−{P(01|00)+P(10|00)+P(11|00)}.

## 5. Simulation Results

This paper considers a uniform linear transmit array consisting of $M_{T}=10$ antenna elements spaced one-half wavelength apart. The purpose of this array is to maximize the directivity of the transmitted signal and minimize interference at the same time. In addition to the radar operation within the main beam, it is assumed that a communication message of two FSK symbols is added cooperatively during each radar pulse. This is performed to develop a composite signal containing four bits of information transmitted through the channel. The baseband signals with FSK modulation are generated using the frequencies $f_{1}=100$ Hz and $f_{2}=200$ Hz in our simulations. The main beam is fixed in a specified direction. All simulations are performed using Matlab 2021a, on a system with an Intel microprocessor Corei5, 11th generation, 8 GB RAM and 2 GB graphics memory.

In the following sections, we will present the simulation results with different illustrative examples for clarity and manageable acquaintance.

### 5.1. Example 1: Single Communication Receiver

**Figure 5:**
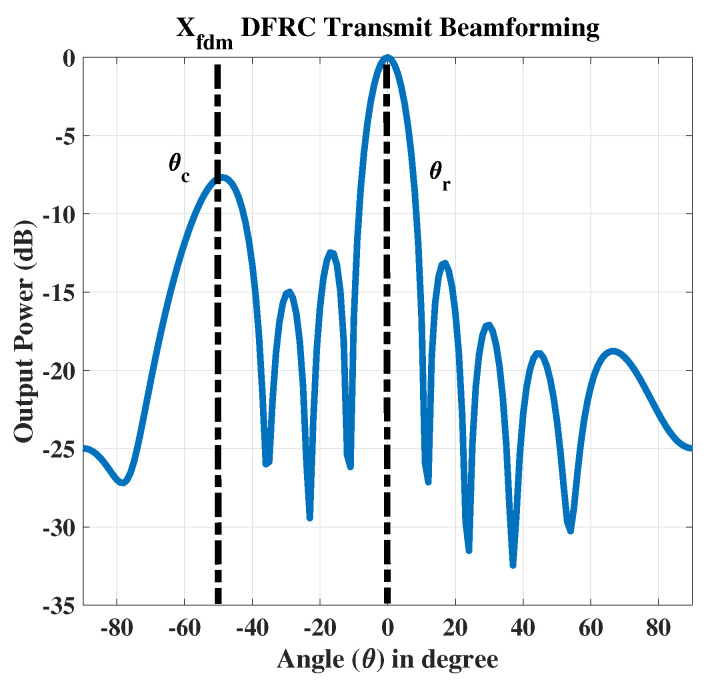
The transmitted signal with radar $\theta_{r}=0^{\circ }$ and the single communication receiver at $\theta_{c}=-50^{\circ }$.

**Figure 6:**
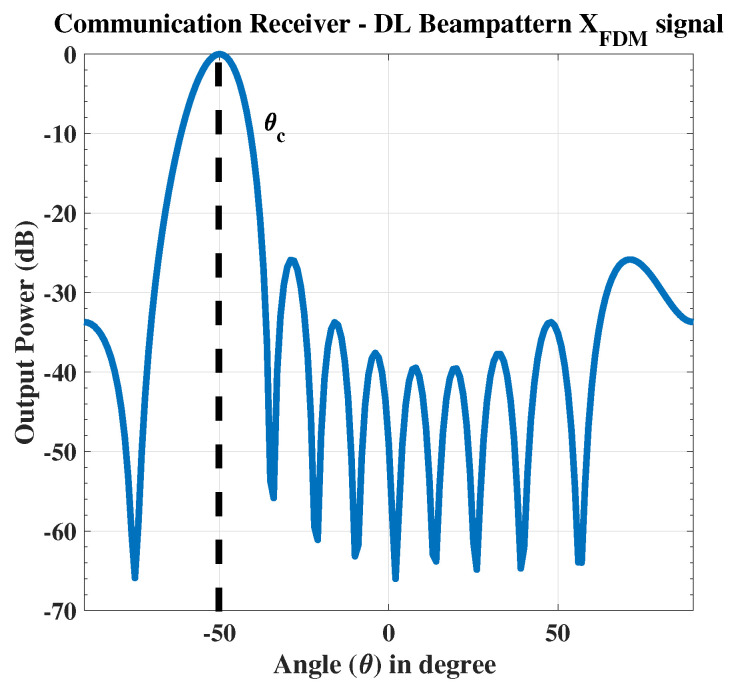
The proposed composite FDM signal received at the communication receiver.

In the 1st case, the radar target is fixed at $\theta_{r}=0^{\circ }$ while the communication receiver is placed at $\theta_{c}=-50^{\circ }$ as shown in Figure 5. The signal received at the communication receiver is shown in Figure 6.

### 5.2. Example 2: Multiple Communication Receiver

**Figure 7:**
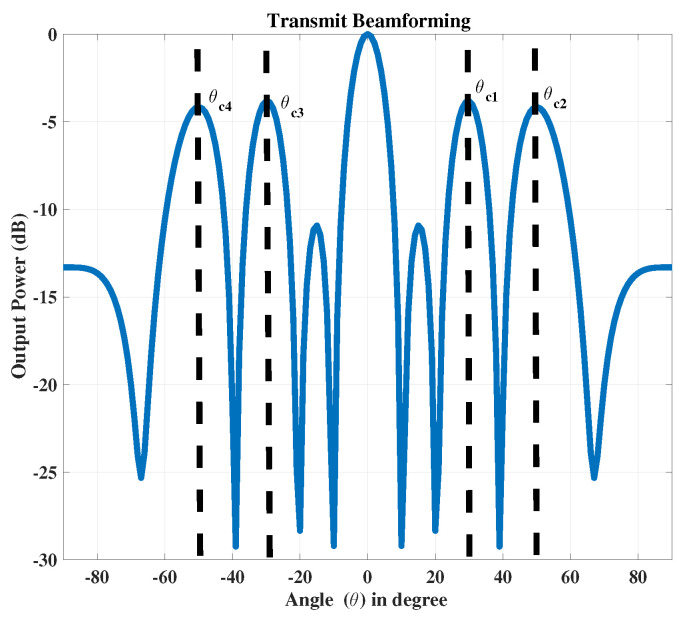
The transmitted signal with the single radar receiver at $\theta_{r}=0^{\circ }$ and the four communication receivers at $\theta_{c1}=30^{\circ }$, $\theta_{c2}=50^{\circ }$, $\theta_{c3}=-30^{\circ }$ and $\theta_{c4}=-50^{\circ }$.

**Figure 8:**
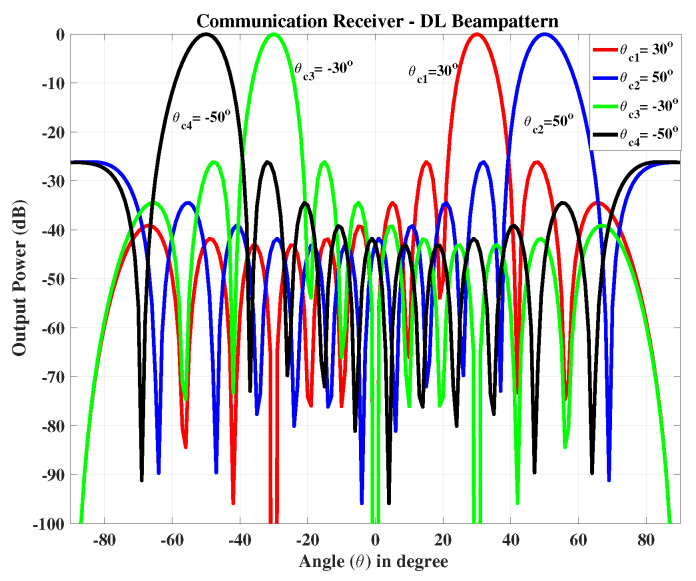
The communication receiver at $\theta_{c1}=30^{\circ }$, $\theta_{c2}=50^{\circ }$, $\theta_{c3}=-30^{\circ }$ and $\theta_{c4}=-50^{\circ }$.

At the communication receiver, we have $N_{R}=10$ antenna elements with same arrangements as used for radar receiver. In the 2nd case we only consider one radar target which lies in the main beam fixed at $\theta_{r}=0^{\circ }$, while four communication receivers are located in the side lobes at $\theta_{c1}=30^{\circ }$, $\theta_{c2}=50^{\circ }$, $\theta_{c3}=-30^{\circ }$ and $\theta_{c4}=-50^{\circ }$, as shown in Figure 7. The data is transmitted to the communication receivers using the broadcast mode. The signal received at each communication receiver is shown in Figure 8.

### 5.3. Bit Error Rate Comparison of the Proposed Scheme

In this study, we assume that the average transmit power of each transmit antenna is normalized to 1, i.e., the total transmit power is fixed to $P_{total}=1$. For each method considered, the total transmitted power is distributed evenly among the number of waveforms. To calculate the BER, $10^{6}$ pulses are considered in the embedding process. As a result, the performance of the various methods can be compared objectively and fairly. This ensures that the methods are evaluated based on the same SNR and that the average power of each transmit antenna remains the same.

**Figure 9:**
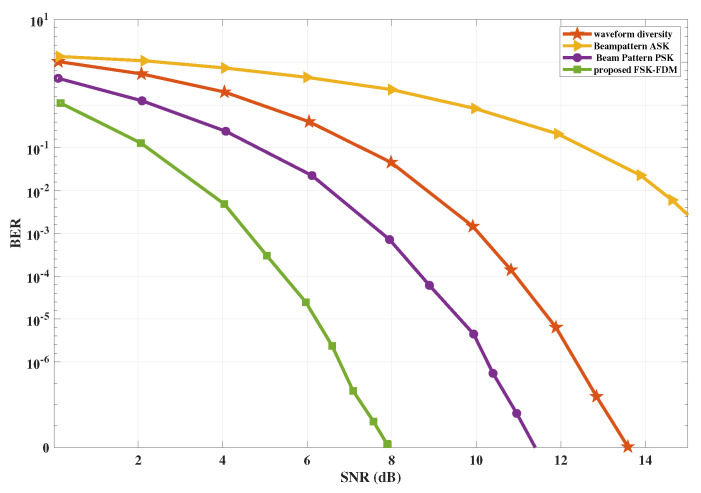
The performance comparison of the proposed scheme with existing schemes.

The performance of both cases explained in examples 1 and 2 of the proposed scheme remains the same, and no degradation is observed. For the case of four communication receivers as shown in example 2, there is a slight degradation in the BER compared to the single communication receiver due to interference. We compared the performance of the proposed scheme for a single communication receiver with side lobe control and waveform diversity cited in [29] and achieved improved BER performance. Moreover, the proposed scheme outperforms the ASK-based information embedding technique as cited in [27]. It can also be observed from the results shown in Figure 9, that the proposed scheme converges more quickly with the safe margin of 3dB when compared with the beam pattern PSK-based approach [26] in terms of BER and SNR.

### 5.4. Decoding Information Bits

**Figure 10:**
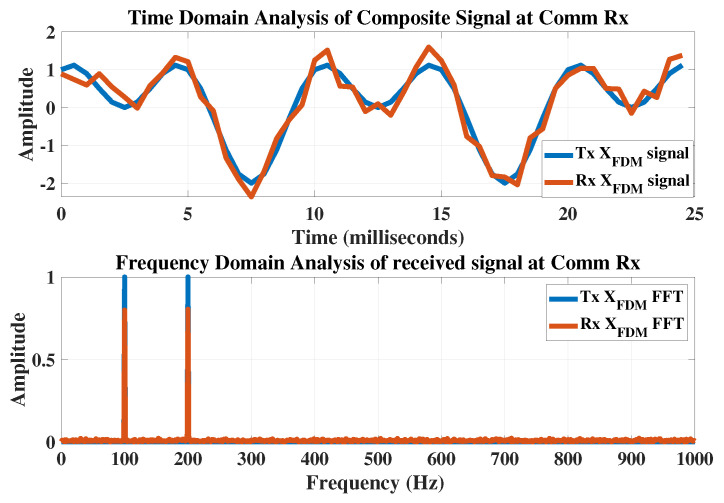
The performance comparison of the transmitted and received FDM signal and their frequency response.

All communication receivers are assigned the same weight vectors. The lookup table is shared with all communication receivers in advance. The shape of the composite signal at the transmitted and received side is shown in Figure 10.

**Figure 11:**
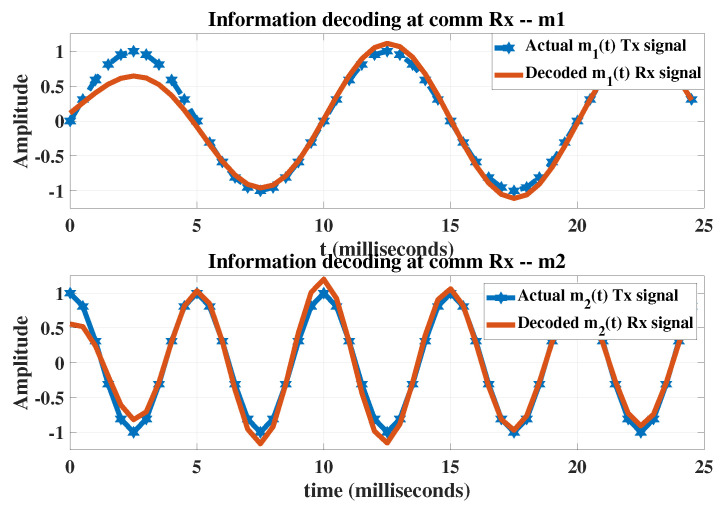
The information decoding at the communication receiver after filtering.

The FFT analysis reveals that both the transmitted and received signals have the same frequency content. Similarly, in Figure 11, each bit is correctly decoded after filtering.

### 5.5. Example 3: Security of Communication Process

**Figure 12:**
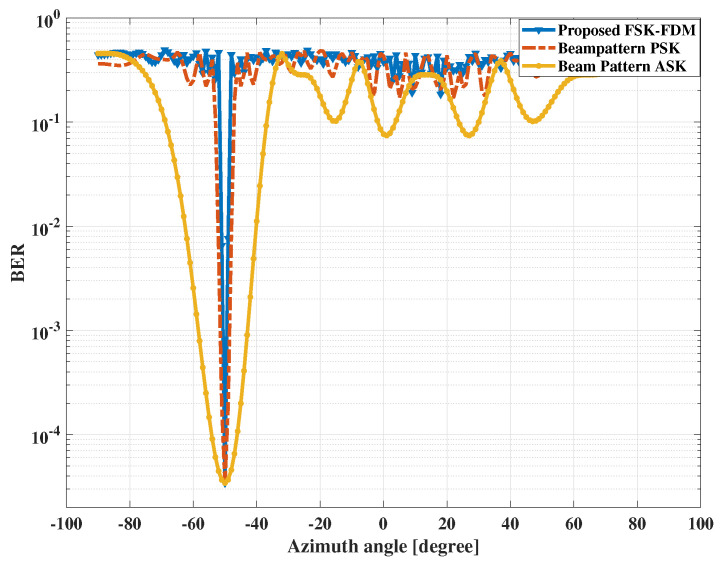
The performance comparison against the intercepts at the communication receiver at $\theta_{c}=-50^{\circ }$.

In this section, we discuss the concerns related to the security in communication. We calculated the SNR for all angles used for transmission. It is clear from Figure 12 that we have minimum interference at the desired angle, i.e., $\theta_{c}=-50^{\circ }$ while, the rest of the angles suffer very high levels of interference. The total number of bits transmitted was $10^{6}$. The SNR was fixed at 5 dB. The results are compared with beam pattern ASK- and beam pattern PSK-based approaches. It is concluded that the performance of the proposed scheme is better than these methods at the same SNR.

### 5.6. DoA Estimation Performance

**Figure 13:**
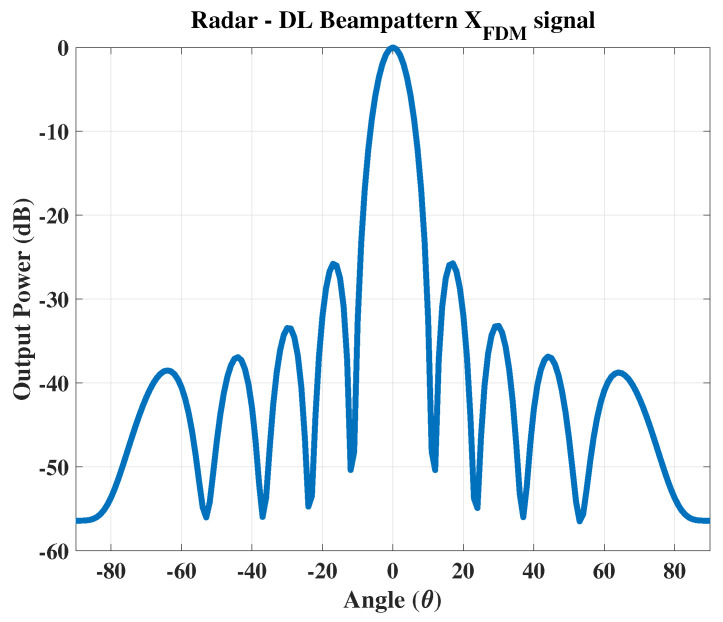
The proposed composite FDM signal received at the radar receiver.

Our objective in this section is to assess the accuracy of DoA estimation for radar operation. A target is assumed to be located in the far field region at a distance of $\theta_{r}=0^{\circ }$. It is assumed that the target reflection coefficients remain constant from pulse to pulse during the radar pulse period but will change pulse-to-pulse as they are drawn from a normal distribution. The number of radar receiver array elements is set to $M_{R}=10$. The number of pulses used was N=100, and 100 snapshots per pulse were used at the radar receiver to build the data covariance matrix. Bartlett beam-forming is used to estimate the DoA for all methods tested. Throughout the different scenarios, i.e., communication in the main and side lobes, no performance degradation was observed in terms of radar operation and DoA estimation. The results are presented in the form of power vs. SNR, as shown in the Figure 13.

### 5.7. Example 4: Probability of Target Detection

In this subsection, we discuss the probability of target detection at different SNR levels. A single target is considered in this case, located at $\theta_{r}=0^{\circ }$ with $M_{R}=10$ antenna elements. The receiver operating characteristic (ROC) curves are calculated between the probability of detection $P_{d}$ and the probability of a false alarm $P_{FA}$ at different SNR levels.

Mathematically, ROC can be calculated as (12)

(12) $$P_{d}=\frac{1}{2}\left[erfc\left(erfc^{-1}\left(2P_{FA}\right)-\sqrt{\chi }\right)\right].$$

where χ is the SNR.

**Figure 14:**
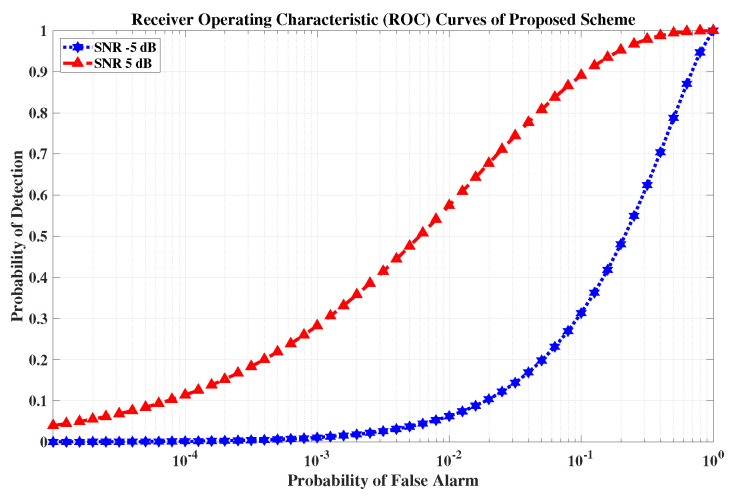
The receiver operating characteristic curves of the proposed scheme.

Figure 14 shows the detection probability at an SNR of −5 dB and 5 dB. Finally, it is observed that embedding information into the radar emission does not affect radar operation.

A comparison of the proposed technique with other existing techniques in terms of BER vs. SNR are provided in Table 3.

A total of $10^{6}$ bits were transmitted. The security against intercepts is presented in Table 4. As can be seen in the table, the proposed technique outperforms the other techniques.

Implementing an FSK–FDM-based DFRC transmitter requires special attention to the SNR since it directly impacts the data rate. Based on simulation results, it is clear that the proposed scheme performs well at higher SNR values. Moreover, we have addressed jamming and the computational complexity of the system at the communication and radar receivers. The proposed system achieves a better trade-off between jamming robustness and data rate than other systems. It also has lower computational complexity, making it a cost-effective solution. Finally, it offers greater flexibility in terms of modulation format and data rate.

## 6. Conclusions and Future Work

A novel approach to a dual-function radar communication system was introduced in the paper. The proposed technique increased the data rate for the communication receiver. Moreover, it delivered information to the communication receivers with an improved BER regardless of their location in the main or side lobes of the radar beam. Next, orthogonal frequency and waveform-based combinations were used to transmit binary information to each communication user, facilitating double the PRF-based data rate with improved secrecy. Finally, diverse scenarios were considered and extensive simulations were conducted to validate the effectiveness of the proposed technique. Currently, only one waveform is considered using likelihood-based estimation for DFRC. Keeping all constraints, such as inter-symbol interference and SNR vs. BER, more than one waveform shall be considered using FDM techniques. In addition, a different scenario needs to be developed when the radar lies in the side lobe, and communications are in the main lobe. In addition, an algorithm needs to be developed to decide when ASK-based techniques should be used and which scenario will work well with PSK-based techniques.

## Figures and Tables

**Table 1:** The lookup table.

$m_{1}\left(t\right)=\xi_{00}\left(t\right)=\psi_{0,f_{0}}\left(t\right)$	$m_{2}\left(t\right)=\xi_{01}\left(t\right)=\psi_{0,f_{1}}\left(t\right)$
$m_{3}\left(t\right)=\xi_{10}\left(t\right)=\psi_{1,f_{0}}\left(t\right)$	$m_{4}\left(t\right)=\xi_{11}\left(t\right)=\psi_{1,f_{1}}\left(t\right)$

**Table 2:** Random information bit mapping to a JWF combination.

…	0	1	1	1	1	0	0	0	…
	$\xi_{01}\left(t\right)$		$\xi_{11}\left(t\right)$		$\xi_{10}\left(t\right)$		$\xi_{00}\left(t\right)$		

**Table 3:** The effectiveness of the proposed technique over existing techniques in terms of BER vs. SNR.

	Waveform	Beam Pattern	Beam Pattern	FSK–FDM
SNR	Diversity	ASK	PSK	(Proposed)
0	912	913	862	804
2	872	903	809	711
4	830	886	738	568
6	760	864	635	3391
8	665	835	485	007
10	516	791	264	003

**Table 4:** The effectiveness of the proposed technique over existing techniques in terms bit error rate vs. security against intercept at the communication receiver (beam width in degrees) for $\theta_{c}=50^{\circ }$.

	Waveform	Beam Pattern	Beam Pattern	FSK–FDM
BER	Diversity	ASK	PSK	(Proposed)
$10^{-1}$	34	32	6	4
$10^{-2}$	24	22	4	3
$10^{-3}$	17	15	3	1.5
$10^{-4}$	10	8	1.5	1

## Data Availability

Not applicable.
